# Genomic insights into one carbapenem-resistant and multidrug-resistant *Proteus mirabilis* strain harboring chromosome-borne *bla*_OXA-23_

**DOI:** 10.1128/spectrum.03438-25

**Published:** 2026-07-17

**Authors:** Dexing Qiu, Limei Zhang, Yanju He, Yi Zhang, Jing Yang, Mingxiao Chen, Xiaobin Li, Yuqing Weng

**Affiliations:** 1 Guangdong Provincial Key Laboratory of Tumor Interventional Diagnosis and Treatment, Zhuhai People’s Hospital (The Affiliated Hospital of Beijing Institute of Technology, Zhuhai Clinical Medical College of Jinan University) 198044, Zhuhai, China; 2 Department of Pulmonary and Critical Care Medicine, Zhuhai People's Hospital (The Affiliated Hospital of Beijing Institute of Technology, Zhuhai Clinical Medical College of Jinan University) 198044, Zhuhai, China; 3 Clinical Laboratory Medicine Department, The Second Affiliated Hospital of Guangzhou Medical University, Guangzhou, China; Newcastle University, Newcastle Upon Tyne, United Kingdom

**Keywords:** carbapenem-resistant *Proteus mirabilis*, chromosomally located *bla*_OXA-23_, multidrug resistance, genetic profiles, whole-genome sequencing

## Abstract

*Proteus mirabilis* is an emerging multidrug-resistant (MDR) pathogen capable of causing severe nosocomial infections. This study characterizes a MDR and carbapenem-resistant *P. mirabilis* strain 20119 isolated from the respiratory tract of a pneumonia inpatient in China. A systematic investigation of antibiotic resistance genes (ARGs) and their genetic environments was conducted. Whole-genome sequencing (WGS) revealed the chromosomal carriage of *bla*_OXA-23_, a gene encoding a class D carbapenemase, within a Tn*2006* composite transposon flanked by IS*Aba1* elements. Global biogeographic analysis of the *P. mirabilis* strains with a complete genome indicated that Tn*2006*-associated *bla*_OXA-23_-harboring *P. mirabilis* strains were predominant in Asia, while Tn*2008*-associated *bla*_OXA-23_-harboring *P. mirabilis* strains were prevalent in Europe. In order to clarify the genetic contexts and mechanisms underlying *bla*_OXA-23_-mediated carbapenem resistance and other ARGs, we categorized the major genetic backdrop associated with chromosomally located ARGs in *P. mirabilis* strain 20119 into five sections: (i) the carbapenem-resistant region harboring *bla*_OXA-23_; (ii) the resistance structure “IS*Ecp1-bla*_CTX-M-14_-IS*903B/*∆IS*903B*”; (iii) the MDR region harboring *mph(E*), *msr(E*), *armA*, and *sul1;* (iv) the prophage containing *bla*_DHA-1_ *and qnrB4;* and (v) the Tn*7* class two integron harboring *aadA1*, *sat2,* and *dfrA1*.

**IMPORTANCE:**

The dissemination of carbapenem-resistant *P. mirabilis* poses a severe clinical threat owing to constrained effective therapeutic alternatives. This work is significant, as it provides the first complete genomic identification of a *bla*_OXA-23_-harboring human-derived *P. mirabilis* in China and the first worldwide isolate from the respiratory tract with a complete genome. The *P. mirabilis* strain 20119 exhibited an MDR profile, harboring additional resistance genes (*bla*_CTX-M-14_, *bla*_DHA-1_, *armA*, etc.) clustered within mobile genetic elements (MGE, e.g., IS*Ecp1*, IS*26*, prophage, and class 2 integron), facilitating horizontal transfer. This study focuses on describing the genomic contexts of the five resistance regions co-occurring with *bla*_OXA-23_ and other ARGs. This study underscores the emergence of *P. mirabilis* as a concerning reservoir for *bla*_OXA-23_ and other ARGs and highlights the fundamental contribution of MGEs to horizontal gene transfer (HGT).

## INTRODUCTION

*Proteus mirabilis* is a rod-shaped, gram-negative, facultatively anaerobic, zoonotic pathogen. It is classified within the order Enterobacterales and the family *Morganellaceae* (1). This opportunistic pathogen causes increasingly prevalent and severe nosocomial infections (2), most notably systemic infections documented in the respiratory tract, bloodstream, wounds, and urinary tract, often in polymicrobial contexts (3–5), with additional zoonotic and environmental transmission risks (6). The success of *P. mirabilis* as a significant clinical threat appears to be associated with its significant resistance to antibiotics. Critically, beyond its intrinsic resistance to tetracyclines and polymyxins, the expanding burden of *P. mirabilis* infections necessitates heightened focus on acquired antibiotic resistance genes (ARGs) (7). *P. mirabilis* isolates have been documented to carry a suite of ARGs encoding ESBLs (e.g., TEM, SHV, CTX-M, VEB, and PER) (8, 9), acquired cephalosporinases (e.g., DHA, CMY, FOX, and ACC) (8, 10), and carbapenemases (e.g., KPC, VIM, IMP, NDM, and OXA-48-like) (11, 12). *P. mirabilis* is increasingly associated with various carbapenemase genes (13).

Although relatively few class D enzymes demonstrate significant carbapenemase activity, certain variants have become clinically predominant in specific pathogens (14). The OXA-48-like enzymes are most often reported in *Klebsiella pneumoniae*, *Escherichia coli,* and *Enterobacter cloacae* (12), while OXA-23, OXA-24, OXA-40, OXA-51, OXA-58, and OXA-143 are typically associated with *Acinetobacter baumannii* (13, 15). Notably, OXA-23—the most widespread carbapenem-hydrolyzing class D enzyme (CHDL) (16)—was originally restricted to *Acinetobacter* spp. after its initial identification in 1985 (17), and it can be found in a variety of composite transposons, including Tn*2006*, Tn*2007*, Tn*2008*, and Tn*2009* (18). However, since its first report in *P. mirabilis* (19), OXA-23 has been increasingly detected in *Proteus* species (13), indicating successful cross-genus transmission and potential genomic adaptation that may complicate control efforts. These β-lactamase-encoding genes often co-occur with other resistance determinants. ESBLs and AmpC genes are key drivers behind resistance to third-generation cephalosporins and propel the surge of MDR and extensively drug-resistant (XDR) phenotypes (20, 21), positioning *P. mirabilis* among the WHO’s highest-priority pathogens requiring urgent research and development efforts (22). Critically, the ARGs carried by *P. mirabilis* disseminate via MGEs located on chromosomes or plasmids. Typically, *bla*_OXA-23_ and adjacent insertion sequences (ISs), including IS*Aba1*, are located on composite transposons Tn*2006* and Tn*2008*, along with other ARGs alongside ISs (e.g., *IS26*, IS*Ecp1*, and IS*903B*), class 1/2 integrons, prophages, and resistance islands that facilitate stable HGT across strains and species.

In this study, WGS was conducted on OXA-23-producing MDR *P. mirabilis* for the identification of antimicrobial resistance profiles and genomic features. We focused on clarifying the association between the chromosome-borne *bla*_OXA-23_ and MGEs and evaluated its potential for horizontal gene transfer (HGT).

## RESULTS

### Identification and antimicrobial susceptibility of *P. mirabilis* strain 20119

The *P. mirabilis* strain 20119 was obtained from the sputum of a 78-year-old inpatient suffering from severe pneumonia in February 2024. It was identified as *P. mirabilis* using the VITEK-2 Compact system, and this result was verified through 16S rRNA gene sequencing.

The results of the antimicrobial susceptibility test determined by the broth microdilution method revealed that *P. mirabilis* strain 20119 exhibited resistance to carbapenems (imipenem), cephalosporins (cefuroxime, cefuroxime axetil, and ceftriaxone), quinolones (levofloxacin), and β-lactam/β-lactamase inhibitor combinations (piperacillin/tazobactam) (Table 1). Additionally, *P. mirabilis* strain 20119 was characterized by intermediate resistance to aminoglycosides (amikacin) (Table 1).

**TABLE 1:** Minimum inhibitory concentration (MIC) values of *P. mirabilis* strain 20119^*a*^

Antibiotics		MIC (μg/mL)	Interpretation
Categories	Name		
Cephalosporins	Cefuroxime	≥64	R
	Cefuroxime axetil	≥64	R
	Cefoxitin	≤4	S
	Ceftriaxone	32	R
	Ceftazidime	≤0.12	S
	Cefepime	2	S
Carbapenems	Imipenem	4	R
Quinolones	Levofloxacin	≥8	R
Aminoglycosides	Amikacin	32	I
Sulfonamides	Trimethoprim/ sulfamethoxazole	40	S
β-lactam/β-lactamase inhibitor combinations	Piperacillin/tazobactam	32	R

^*a*^ S, susceptible; R, resistant; I, intermediate.

### Genomic characteristics of *P. mirabilis* strain 20119

The fully sequenced genome of *P. mirabilis* strain 20119 comprises a ~4.1 Mb chromosome (GenBank accession CP169048) and a ~38.7 kb plasmid (GenBank accession CP169049).

The results of Abricate showed that all the ARGs were only distributed on the chromosome of *P. mirabilis* strain 20119, including the carbapenem gene (*bla*_OXA-23_), ESBL gene (*bla*_CTX-M-14_), AmpC β-lactamase gene (*bla*_DHA-1_), as well as ARGs conferring resistance to aminoglycosides (*armA* and *aadA1*), macrolides (*msr(E)-mph(E*)), quinolones (*qnrB4*), sulfonamides (*sul1*), trimethoprim (*dfrA1*), tetracycline (*tet(J)-tetR(J*)), and phenicols (*cat*). Notably, *P. mirabilis* strain 20119 represents the first documented *bla*_OXA-23_-positive *P. mirabilis* with a complete genome in China. Together with the other seven human-derived strains from different countries carrying *bla*_OXA-23_ with complete genomes, this finding suggests the global spread potential of OXA-23-positive *P. mirabilis*.

It is notable that these resistance genes were usually surrounded by ISs, including families IS*3*, IS*4*, IS*5*, IS*6*, IS*26,* and IS*256*. Based on their genetic locations, chromosomally encoded ARGs were classified into five distinct antibiotic resistance regions: (i) the carbapenem-resistant region harboring *bla*_OXA-23_; (ii) the resistant structure “IS*Ecp1-bla*_CTX-M-14_-IS*903B/*∆IS*903B*”; (iii) the MDR harboring *mph(E*), *msr(E*), *armA*, and *sul1*; (iv) the prophage containing *bla*_DHA-1_ and *qnrB4*; and (v) the Tn*7* class 2 integron harboring *aadA1*, *sat2*, and *dfrA1*.

### The carbapenem-resistant region harboring *bla*_OXA-23_

**Fig 1:**
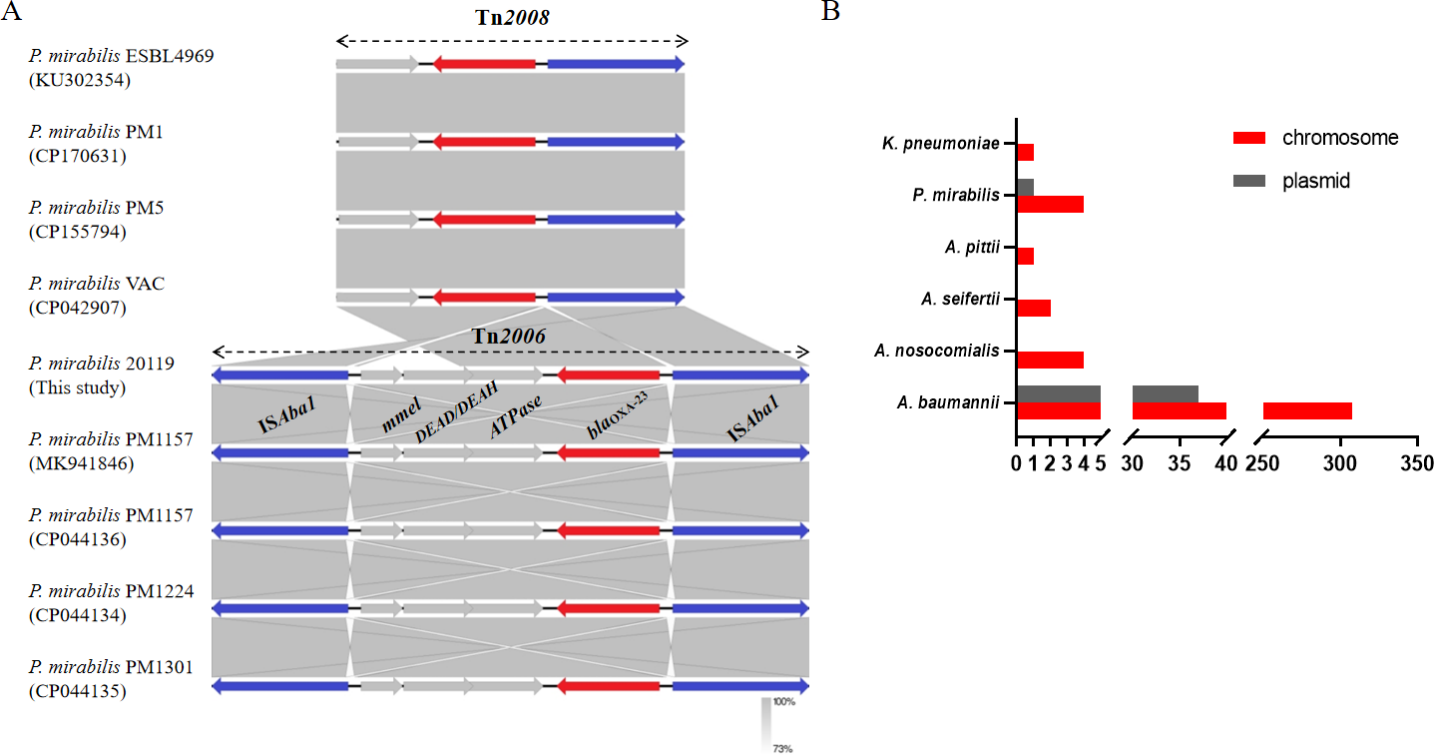
Genetic structures of the carbapenem-resistance region harboring *bla*_OXA-23_. (**A**) Comparative analysis of genetic structures of the carbapenem-resistance region associated with *bla*_OXA-23_ in *P. mirabilis*. The sequences used for comparison were linked to the following GenBank accession numbers: ESBL4969 chromosome (KU302354), PM1 chromosome (CP170631), PM5 chromosome (CP155794), VAC chromosome (CP042907), 20119 chromosome (CP169048), PM1157 plasmid pOXA-23 (MK941846), PM1157 chromosome (CP044136), PM1224 chromosome (CP044134), and PM1301 chromosome (CP044135). Genes and ORFs are shown as arrows, and the direction of transcription is indicated by arrowheads. Shared regions with 99% query cover and 99% identity. Resistance and transposase (ISs) genes are shown in red and blue, respectively. (**B**) An overview of the human-derived strains harboring the Tn*2006*.

The carbapenemase gene (*bla*_OXA-23_) (coordinates: 179,468–184,236) was located in the ~4.6 kb composite transposon Tn*2006* bordered by two inversely oriented copies of IS*Aba1*. Specifically, one copy was located upstream and was sharing the same transcriptional orientation as *bla*_OXA-23_, preceding the restriction-modification enzyme gene *mmeI*, DEAD-DEAH box helicase-like gene, and ATPase gene. The second IS*Aba1* is positioned downstream in the opposite orientation to *bla*_OXA-23_ (Fig. 1A).

BLAST analysis of *P. mirabilis* identified eight human-derived strains carrying the *bla*_OXA-23_ gene. Of these, six were isolated from urine, one from a rectal swab collected in France, and one from sputum as described in this work. The *bla*_OXA-23_ gene was located on the chromosome and mediated by Tn*2006* in three isolates, on both the chromosome and plasmid associated with Tn*2006* in one isolate, and within Tn*2008* on the chromosome in the remaining four isolates (Fig. 1A). Tn*2008*-associated strains were predominantly found in European countries—Finland, Switzerland, and France. In contrast, Tn*2006*-carrying strains were largely detected in Asia, specifically in Singapore and China (Table 2). In addition, we identified 368 human-derived strains (coverage ≥99% and identity ≥99%) harboring Tn*2006. Acinetobacter* spp. were identified as the primary reservoirs, while sporadic detection of Tn*2006* was observed within the family *Morganellaceae*, particularly in *P. mirabilis* (Fig. 1B).

**TABLE 2:** Clinical features of OXA-23-producing *P. mirabilis* isolates

Isolates	ST	Source, sample	Location	Country	Year of isolation	Reference	GenBank accession numbers
ESBL4969	–^*a*^	Human, Urine	Tn2008, chromosome	Finland	2014	(23)	KU302354
PM1	ST142	Human, Urine	Tn2008, chromosome	Switzerland	2017	(24)	CP170631
PM5	ST142	Human, Urine	Tn2008, chromosome	Switzerland	2023	(24)	CP155794
VAC	ST142	Human, Rectal swab	Tn2008, chromosome	France	2016	(25)	CP042907
20,119	ST135	Human, Sputum	Tn2006, chromosome	China	2024	This study	CP169048
PM1157	ST192	Human, Urine	Tn2006, Plasmid; Tn2006, chromosome	Singapore	2014	(26)	MK941846; CP044136
PM1224	ST192	Human, Urine	Tn2006, chromosome	Singapore	2014	(26)	CP044134
PM1301	ST193	Human, Urine	Tn2006, chromosome	Singapore	2014	(26)	CP044135

^*a*^ –, no data.

### The resistant structure “IS*Ecp1-bla*_CTX-M-14_-IS*903B/*∆IS*903B*” in *P. mirabilis* strain 20119

**Fig 2:**
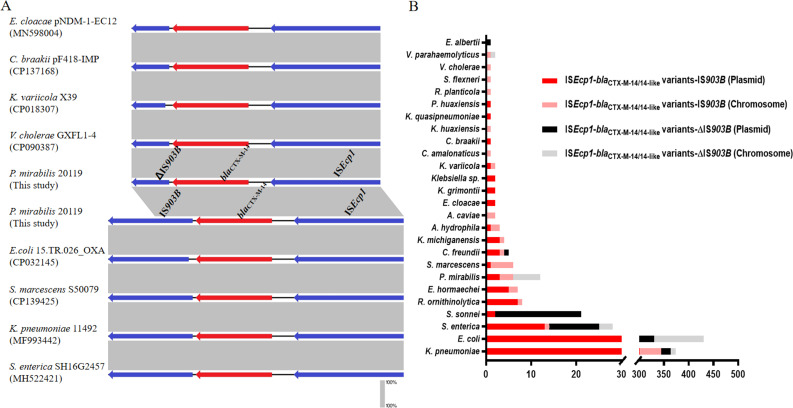
Genetic structures of the resistant structure “IS*Ecp1-bla*_CTX-M-14/14-like_ variants-IS*903B/*∆IS*903B.*” (**A**) Comparative analysis of genetic structures of the “IS*Ecp1-bla*_CTX-M-14/14-like_ variants-IS*903B/*∆IS*903B.*” The sequences used for comparison were linked to the following GenBank accession numbers: EC12 plasmid pNDM-1-EC12 (*E. cloacae*, MN598004), F418 plasmid pF418-IMP (*C. braakii*, CP137168), X39 chromosome (*K. variicola*, CP018307), GXFL1-4 chromosome (*V. cholerae*, CP090387), 20119 chromosome (*P. mirabilis*, CP169048), 15.TR.026_OXA chromosome (*E. coli*, CP032145), S50079 chromosome (*S. marcescens*, CP139425), S50079 plasmid unnamed (*S. marcescens*, MF993442), and SH16G2457 plasmid pSH16G2457 (*S. enterica*, MH522421). Genes and ORFs are shown as arrows, and the direction of transcription is indicated by arrowheads. Shared regions with 99% query cover and 99% identity. Resistance and transposase (ISs) genes are shown in red and blue, respectively. (**B**) An overview of the strains harboring the structure “IS*Ecp1-bla*_CTX-M-14/14-like_ variants-IS*903B*/∆IS*903B.*”

This study identified two copies of the ESBLs gene *bla*_CTX-M-14_ (coordinates: 1,877,839-1,880,692 and 2,930,249-2,933,639) within IS*Ecp1*-IS*903B*-mediated transposition units. The first copy exhibited the structure IS*Ecp1-bla*_CTX-M-14_-ΔIS*903B* (downstream truncated IS*903B*), while the second displayed a co-directional tandem arrangement IS*Ecp1-bla*_CTX-M-14_-IS*903B* (downstream intact IS*903B*) (Fig. 2A).

A BLAST analysis based on this region was performed in the GenBank database with coverage ≥99% and identity ≥99%. Given that *bla*_CTX-M-14_ shares high similarity with certain *bla*_CTX-M_ variants, differing only by a few nucleotide mutations that result in minimal amino acid changes, the BLAST-based search results for “IS*Ecp1-bla*_CTX-M-14_-ΔIS*903B/*IS*903B*” may encompass not only *bla*_CTX-M-14_ but also closely related *bla*_CTX-M-14/14-like_ variants. To accurately reflect this inclusivity in descriptive statistical reporting, we denote the group as “IS*Ecp1-bla*_CTX-M-14/14-like_ variants-ΔIS*903B/*IS*903B.*” The results revealed that 611 sequences across 25 species contained the intact structure (IS*Ecp1-bla*_CTX-M-14/14-like_ variants-IS*903B*), whereas 308 sequences across eight species harbored the truncated structure (IS*Ecp1-bla*_CTX-M-14/14-like_ variants-ΔIS*903B*). These configurations were disseminated among 20 *Enterobacteriales* species (e.g., *K. pneumoniae*, *E. coli*, *S. enterica*, and *P. mirabilis*). It was noteworthy that both intact and truncated structures were present in the chromosomes and/or plasmids of the 26 species. In *P. mirabilis*, the intact structure was split evenly between the chromosomes and plasmids, while the truncated structure was confined to the chromosomes. Overall, however, plasmids accounted for a higher proportion of these structures (Fig. 2B).

### The MDR associated with *mph(E*), *msr(E*), *armA*, and *sul1* in *P. mirabilis* strain 20119

**Fig 3:**
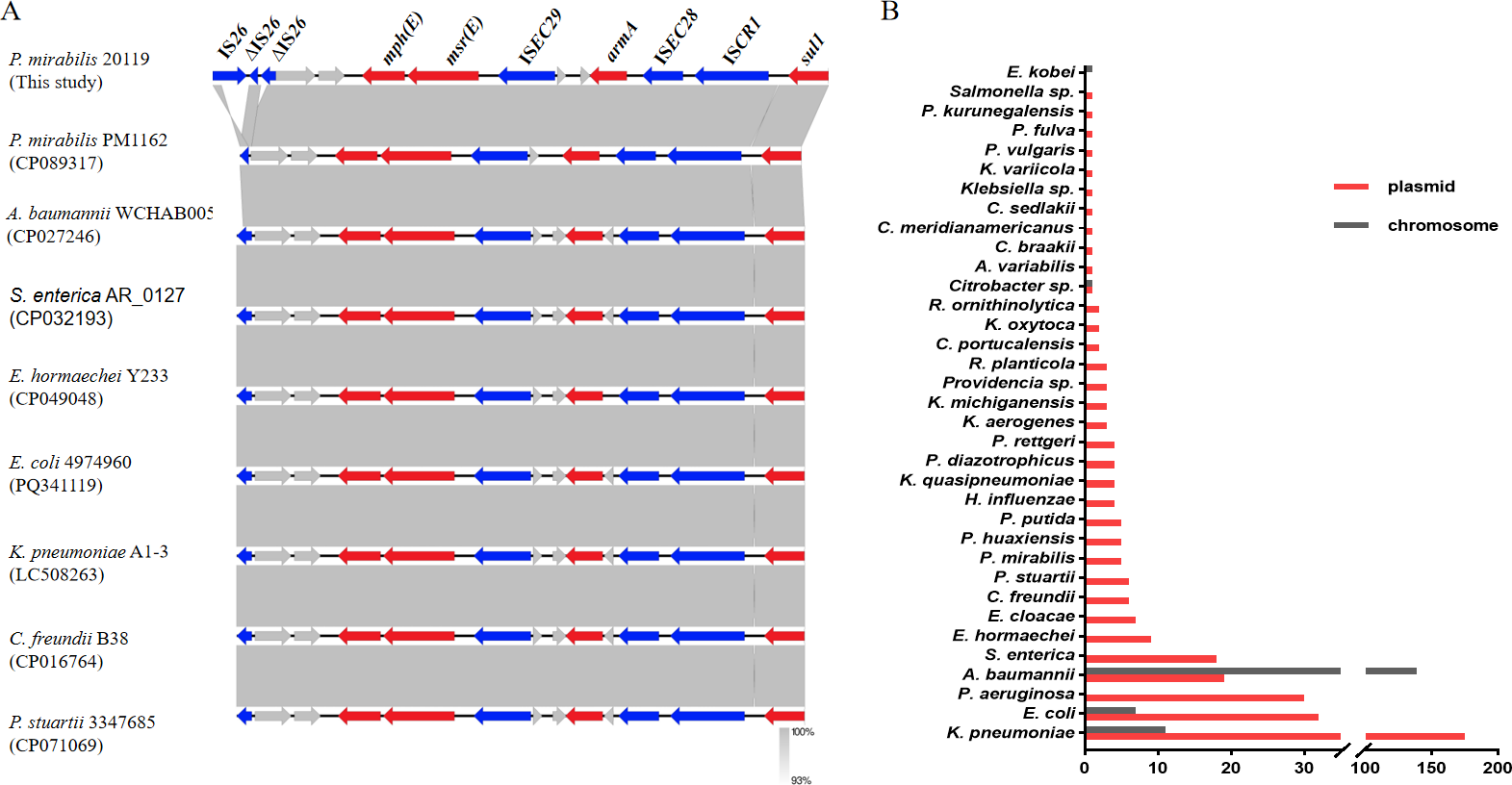
Genetic structures of the MDR region harboring *mph(E*), *msr(E*), *armA,* and *sul*. (**A**) Comparative analysis of genetic structures of the MDR. The sequences used for comparison were linked to the following GenBank accession numbers: 20119 chromosome (*P. mirabilis*, CP169048), PM1162 chromosome (*P. mirabilis*, CP089317), WCHAB005078 chromosome (*A. baumannii*, CP027246), AR_0127 plasmid unnamed2 (*S. enterica subsp*., CP032193), Y233 plasmid p233-142 (*E. hormaechei*, CP049048), 4974960 plasmid unnamed (*E. coli*, PQ341119), A1-3 plasmid pA1-3(*K. pneumoniae*, LC508263), B38 plasmid pOZ181 (*C. freundii*, CP016764), and 3347685 plasmid p3347685_1 (*P. stuartii*, CP071069.1). Genes and ORFs are shown as arrows, and the direction of transcription is indicated by arrowheads. Shared regions with 99% query cover and 99% identity. Resistance and transposase (ISs) genes are shown in red and blue, respectively. (**B**) An overview of the strains harboring the MDR genes.

The ~12.8 kb MDR region of *P. mirabilis* strain 20119 harbors four resistance genes: macrolide resistance gene *mph(E*); macrolide, lincosamide, and streptogramin B resistance gene *msr(E*); aminoglycoside resistance gene *armA*; and sulfonamide resistance gene *sul1*. Genes *armA*, *mph(E*), and *msr(E*) flanked by IS*Ec28* and IS*26*. Upstream of *mph(E*) and *msr(E*) are IS*26*, two truncated IS*26* (∆IS*26*), and replication initiation protein gene, *repM*; downstream lies IS*Ec29*. The armA gene is preceded by IS*Ec29*, a hypothetical protein gene, and an aminomethyltransferase β-barrel domain-encoding gene, while IS*Ec28* and IS*CR1* are located between *armA* and *sul1* (Fig. 3A).

BLAST analysis revealed 521 highly similar sequences (coverage ≥99% and identity ≥99%) across 35 bacterial species, mainly including *P. mirabilis*, *E. coli, K. pneumoniae*, *A. baumannii,* and *Pseudomonas aeruginosa* (Fig. 3B).

### The prophage containing *bla*_DHA-1_*, qnrB4* in *P. mirabilis* strain 20119

**Fig 4:**
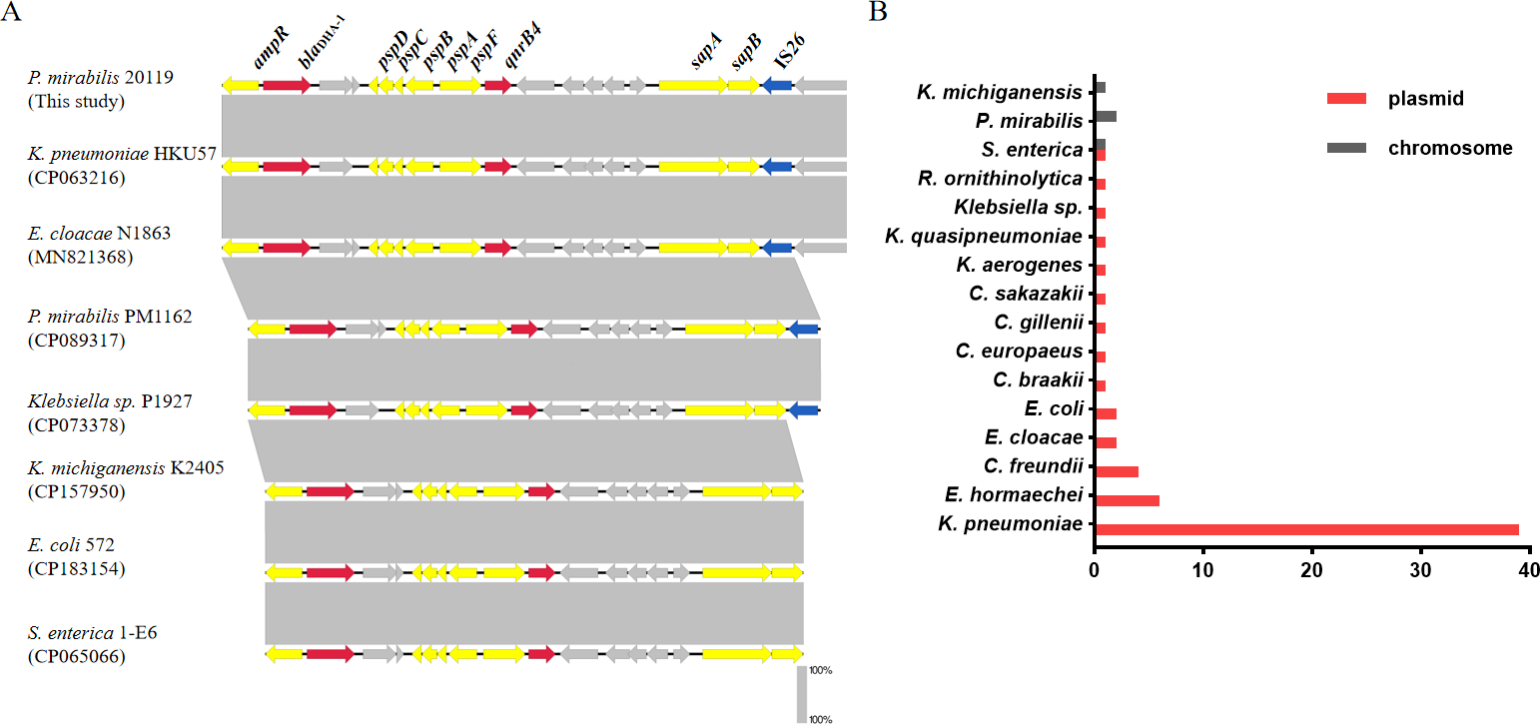
Genetic structures of the prophage containing *bla*_DHA-1_, *qnrB4*. (**A**) Comparative analysis of the genetic structures of the prophage. The sequences used for comparison were linked to the following GenBank accession numbers: 20119 chromosome (*P. mirabilis*, CP169048), HKU57 plasmid (*K. pneumoniae*, CP063216), N1863 plasmid (*E. cloacae*, MN821368), PM1162 chromosome (*P. mirabilis*, CP089317), P1927 plasmid (*Klebsiella sp*., CP073378), *K2405* chromosome (*K. michiganensis,* CP157950), 572 plasmid (*E. coli*, CP183154), and 1-E6 chromosome (*S. enterica*, CP065066). Genes and ORFs are shown as arrows, and the direction of transcription is indicated by arrowheads. Shared regions with 99% query cover and 99% identity. Resistance, transposase (ISs), and prophage genes are shown in red, blue, and yellow, respectively. (**B**) An overview of the strains harboring the prophage containing *bla*_DHA-1_, *qnrB4*.

This ~14.8 kb antibiotic resistance region, characterized as an incomplete prophage, harbors the AmpC gene *bla*_DHA-1_ and the quinolone resistance gene *qnrB4*. The *bla*_DHA-1_ is flanked by the regulator *ampR* upstream and *pspF* (the activator of the *pspABCD* operon) downstream. The *qnrB4* resides downstream of *pspF*, with the region terminating in *sapA*, *sapB*, IS*26*, and a transposase gene (Fig. 4A).

BLAST analysis identified this specific prophage region in 66 sequences (coverage ≥99% and identity ≥99%) from 16 strain species. Most (62/66, 93.94%) occurred on plasmids, predominantly in *K. pneumoniae* and 14 other species. Chromosomal integration was observed in *P. mirabilis* (CP169048, CP089317), *K. michiganensis* (CP157950), and *S. enterica subsp.* (CP065066) (Fig. 4B).

### Tn*7* class 2 integron harboring *aadA1*, *sat2,* and *dfrA1* in *P. mirabilis* strain 20119

**Fig 5:**
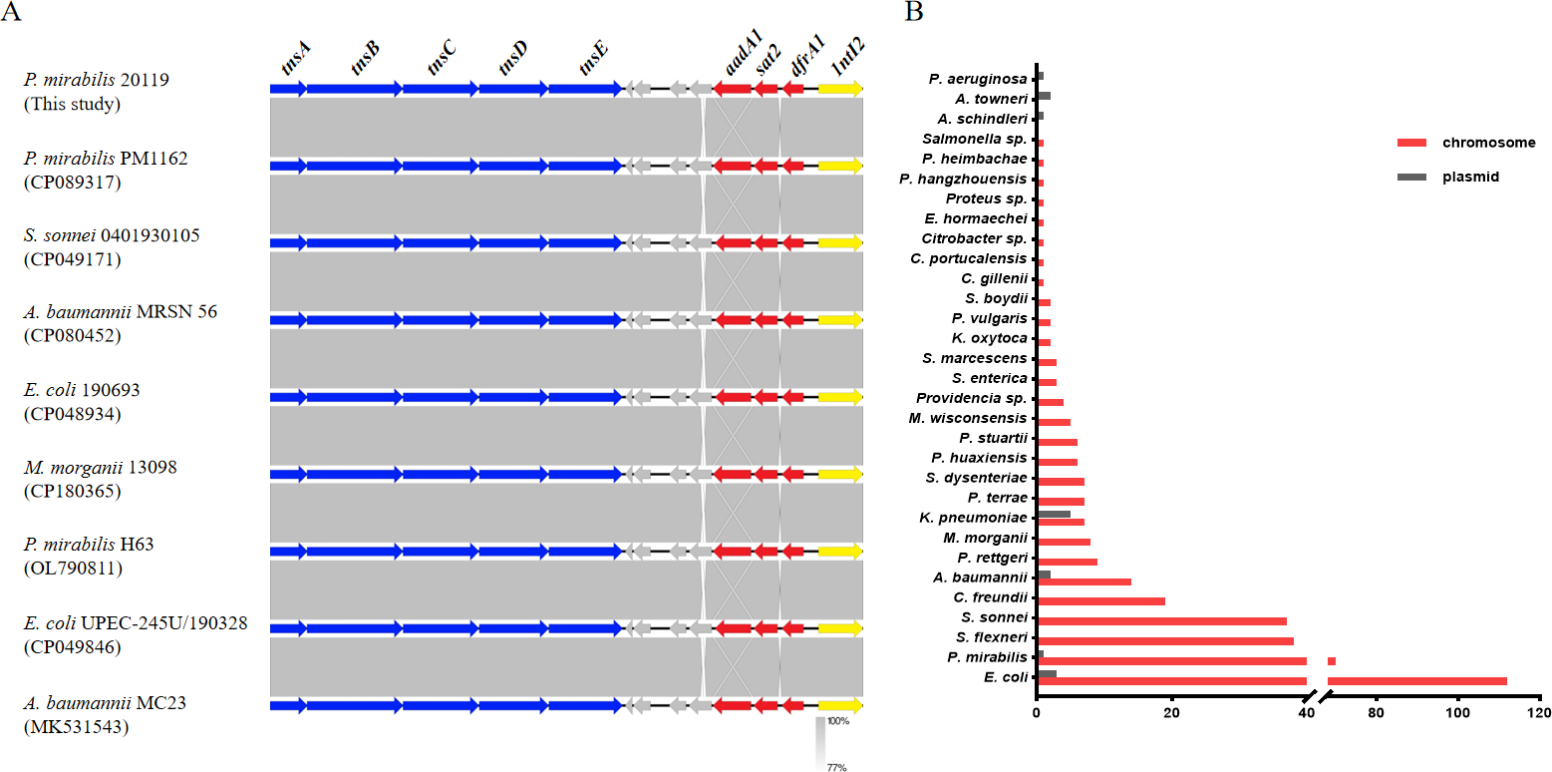
Genetic structures of Tn*7* class 2 integron harboring *aadA1*, *sat2,* and *dfrA1*. (**A**) Comparative analysis of genetic structures of Tn*7* class 2 integron associated with *aadA1*, *sat2,* and *dfrA1*. The sequences used for comparison were linked to the following GenBank accession numbers: 20119 chromosome (*P. mirabilis*, CP169048), PM1162 chromosome (*P. mirabilis*, CP089317), 0401930105 chromosome (*S. sonnei*, CP049171), MRSN 56 chromosome (*A. baumannii*, CP080452), 190693 chromosome (*E. coli*, CP048934), 13098 chromosome (*M. morganii,* CP180365), H63 plasmid (*P. mirabilis*, OL790811), UPEC-245U/190328 plasmid (*E. coli*, CP049846), and MC23 plasmid (*A. baumannii*, MK531543). Genes and ORFs are shown as arrows, and the direction of transcription is indicated by arrowheads. Shared regions with 99% query cover and 99% identity. Resistance, transposase (ISs), and integrase genes are shown in red, blue, and yellow, respectively. (**B**) An overview of the strains harboring Tn*7* class 2 integron associated with *aadA1*, *sat2,* and *dfrA1*.

A ~13 kb class 2 integron was identified in *P. mirabilis* strain 20119. This element harbored three resistance genes: *aadA1* (aminoglycoside), *sat2* (streptothricin), and *dfrA1* (trimethoprim), as well as five transposase genes (*tnsA*, *B*, *C*, *D*, and *E*) and a class 2 integron. They located within a typical transposon Tn*7* that downstream of the transposition-related genes lies a resistance gene cassette (*aadA1-sat2-dfrA1*), beyond which is an open reading frame designated *intI2* (Fig. 5A).

BLAST analysis revealed identical Tn*7* structures (coverage ≥99% and identity ≥99%) in 384 sequences, which the chromosome (369, 96.09%) and plasmid (15, 3.91%), across 31 diverse species, some of them including: *Shigella sonnei*, *E. coli*, *A. baumannii*, *P. mirabilis*, and so on (Fig. 5B).

## DISCUSSION

This work presents the complete genome sequence of *P. mirabilis* strain 20119, a carbapenem-resistance mediated by chromosomal *bla*_OXA-23_ and an MDR isolate from a severe pneumonia patient. Antimicrobial susceptibility testing revealed that only a few drugs—cephalosporins (cefoxitin, ceftazidime, and cefepime) and trimethoprim/sulfamethoxazole—remained active against the MDR *P. mirabilis* 20119 isolate, establishing them as viable therapeutic choices for infections caused by *P. mirabilis*. Notably, epidemiological surveillance reveals that *bla*_OXA-23_ has been detected in diverse clinical specimens, especially in *Acinetobacter* spp. and some strains of Enterobacterales (27). Even *bla*_OXA-23_-harboring carbapenem-resistant clusters have evolved beyond disseminating within and across species (28).

Whole-genome analysis revealed *bla*_OXA-23_ in *P. mirabilis* strain 20119 is carried by Tn*2006*, which is flanked by inversely oriented IS*Aba1* copies. These IS*Aba1* elements provide strong promoter activity, enhancing resistance expression (29). Tn*2006* is one of the most prevalent transposable elements worldwide, as well as Tn*2007*, Tn*2008,* and Tn*2009,* which significantly contribute to *bla*_OXA-23_ dissemination, likely representing primary drivers of global carbapenem resistance (18). In eight OXA-23-producing *P. mirabilis* with complete genome, the *bla*_OXA-23_ were located on both chromosomes and plasmids (23–26). Among them, the geographic distribution of OXA-23 varies by transposon, it is primarily driven by Tn*2008* in Europe, which isolates PM1, PM5, and VAC were all belong to ST142 (24, 25), as well as strain ESBL4969 (GenBank accession number: KU302354) exhibited an equivalent genetic backdrop to the OXA-23-producing *P. mirabilis* reported in the French community (GenBank accession number: SLUF00000000) (23, 30). By Tn*2006* in Asia which the three Singaporean isolates from urine belongs to ST192 and ST193 showed no phylogenetic relatedness to the French community strain (26, 30), while our *P. mirabilis* strain 20119 was the first documented *bla*_OXA-23_-positive *P. mirabilis* with a complete genome in China. It belongs to ST135 and was genetically distinct from all previously reported *bla*_OXA-23_-positive *P. mirabilis* isolates.

The CTX-M ESBLs were first identified in *E. coli* by Alain Philippon from Germany in 1989 (31), and it has developed into more than 200 subtypes subsequently. Typically, the strains harboring CTX-M resistance genes produce ESBLs that are able to bind to the β-lactam ring, a critical antibacterial active structure in cephalosporin drugs, thereby preventing or hindering the binding of antibiotics to their bacterial target sites and leading to multidrug resistance against various cephalosporins (32). This study revealed that *P. mirabilis* strain 20119 carried two copies of the extended-spectrum class A β-lactamase gene *bla*_CTX-M-14_ at distinct genomic locations, which may underlie its resistance to cefuroxime, cefuroxime axetil, ceftriaxone, and other cephalosporins. The *bla*_CTX-M-14_ flanked by insertion sequences IS*Ecp1* and IS*903B*/∆IS*903B* in the same orientation, forming a genetic cassette IS*Ecp1-bla*_CTX-M-14_-IS*903B*/∆IS*903B* (33). This arrangement provides a robust promoter region for the resistance gene, enabling its high expression and facilitating horizontal gene transfer of *bla*_CTX-M-14_ (34).

The MDR region comprising the resistance genes *mph(E*), *msr(E*), *armA,* and *sul1* confers resistance to macrolides, aminoglycosides, and sulfonamide antibiotics. This region features four IS elements: IS*CR1*, IS*Ec28*, IS*Ec29,* and IS*26*. Notably, IS*CR1* facilitates the HGT of *armA* (35). A hybrid promoter can be formed by the −35 consensus sequence (TTGCAA) located in the terminal inverted repeat of IS*26*, provided that it is situated with a 17 bp spacer adjacent to a −10 sequence upstream of the gene (36), urging the combination of IS*26* with the resistance genes to form a composite transposon structure (37). Following the integration of an IS*26* copy, a chromosome or plasmid has an increased propensity to obtain additional adjacent IS26 translocatable units (38). The IS*26* data set established by Song Yuqin et al. also illustrates that a single copy of IS*26* possesses the capability to mediate the random integration and enhance drug resistance of ARGs (39). Therefore, we speculate that this MDR may horizontally disseminate through complex translocatable units mediated by IS*CR1*, IS*Ec28*, IS*Ec29,* and IS*26*. IS*26* can also facilitate the integration of the AmpC β-lactamase resistance gene *bla_DHA-1_* and the quinolone resistance gene *qnrB4* into an incomplete prophage (40), which was predominantly found on plasmids, with only a limited number of instances located on the chromosomes of *P. mirabilis*, *K. michiganensis,* and *S. enterica*. These strains are likely to function as mobile reservoirs for this prophage, enabling the dissemination of resistance through ongoing genetic accumulation and evolutionary processes.

Tn*7*-mediated class 2 integrons were often identified in *P. mirabilis* (41). The stable drug resistance gene cassette *dfrA1-sat2-aadA1*, composed of the trimethoprim resistance gene *dfrA1* and the aminoglycoside resistance gene *aadA1*, cannot be recombined due to an internal stop codon (intI2*), which impairs the ability of this class 2 integron to integrate, recombine, and express resistance. Class 2 integron function can be governed by both external stimuli and the host genome. A key activation mechanism involves mutation of the termination codon, a process whereby integrase activity is restored. This conversion yields functional integrons that confer adaptive advantages for survival (42). The class 2 integron harboring the *dfrA1-sat2-aadA1* cassette is predominantly located on chromosomes, and it has been detected in more than 30 strains.

In summary, *P. mirabilis* strain 20119 represents a high-risk, multidrug-resistant clone. Its resistance profile is driven by a combination of key genes (*bla*_OXA-23_, *bla*_CTX-M-14_, and *armA*) mobilized and amplified by powerful genetic elements like Tn*2006*, IS*Ecp1*, and IS*26*, along with other acquired structures such as a class 2 integron. This study highlights the ongoing evolution and convergence of resistance mechanisms in pathogens, posing a significant challenge for treatment and underscoring the need for vigilant genomic surveillance.

## MATERIALS AND METHODS

### Identification and antimicrobial susceptibility testing of bacterial strains

*P. mirabilis* strain 20119 was obtained from the sputum of a 78-year-old inpatient suffering from severe pneumonia at the Second People’s Hospital of Guangzhou, China, in February 2024. Bacterial identification was performed with the fully automated VITEK-2 Compact system (bioMérieux, France) following the manufacturer’s guidelines. It was further confirmed by 16S rRNA gene sequencing (KmerFinder, SpeciesFinder) (43) that the strain 20119 was *P. mirabilis*.

Susceptibility to antimicrobial agents was determined by the broth microdilution method on the VITEK-2 Compact system. Testing was performed, and the results were annotated following the Clinical and Laboratory Standards Institute (CLSI) M100-S33 (2023) criteria. The following antibacterial agents were used in this test: cephalosporins (ceftazidime, cefepime, cefoxitin, cefuroxime, cefuroxime axetil, and ceftriaxone), carbapenems (imipenem), quinolones (levofloxacin), aminoglycosides (amikacin), sulfonamide (trimethoprim/sulfamethoxazole), and β-lactam/β-lactamase inhibitor combinations (piperacillin/tazobactam).

### Whole-genome sequencing, assembly, and annotation

The WGS of *P. mirabilis* strain 20119 was conducted by BIOZERON Biotechnology Co. Ltd. (Shanghai, China) employing the Illumina NovaSeq 6000 for short-read sequencing and the PacBio Sequel platform for long-read sequencing. Hybrid assembly of the genome was conducted using Unicycler (v0.4.8) (44) with both short and long reads and polishing with Pilon (v1.22) (45). Submission and annotation of the assembled genome for *P. mirabilis* strain 20119 were carried out through the NCBI GenBank platform and its integrated Prokaryotic Genome Annotation Pipeline, respectively (46).

### Genomic bioinformatics analysis of *P. mirabilis* strain 20119

The genetic locations of these acquired ARGs in the genomes of the *P. mirabilis* strain 20119 were confirmed to be on the chromosome through WGS analysis and were further identified using Abricate (https://github.com/tseemann/abricate) with a minimum coverage of 80% and a minimum identity of 80%. The ISs that are adjacent to the ARGs in the strain 20119 genome were identified using ISfinder (47). The GenBank non-redundant database was searched using BLAST to analyze sequence similarity (48), and Easyfig version 2.2.5 (49) was utilized for a vividly visualized comparison of similar sequences. Moreover, ICEfinder (50) was employed to identify chromosomal genomic islands, Phastest (51) was used to detect prophages, and PubMLST (52) served as a platform to determine Sequence Types (STs).

## Data Availability

The complete sequences of the chromosome and plasmid of the *P. mirabilis* strain 20119 were submitted to the GenBank database, under accession numbers CP169048–CP169049.
